# Binary matrices and simplicial complexes: an algebraic-statistics tool to analyze co-activation of electrophysiological signals in cortical cultures

**DOI:** 10.1186/1471-2202-14-S1-P263

**Published:** 2013-07-08

**Authors:** Virginia Pirino, Paolo Massobrio, Eva Riccomagno, Sergio Martinoia

**Affiliations:** 1 Department of Informatics, Bioengineering, Robotics and Systems Engineering, University of Genova, Genova, 16145, Italy; 2 Department of Mathematics, University of Genova, Genova, 16146, Italy

## 

Recent studies [1,2] have shown that a mathematical technique based on simplicial complexes associated with binary matrices that capture relevant data features can be applied to the analysis of electrophysiological data. In this work, we employ this technique to investigate the co-activity level (defined as a period of synchronous firing of a group of neurons) of a neuronal network, both during spontaneous and stimulus-evoked activity conditions. We analyze recordings of cortical neurons coupled to Micro-Electrode Arrays (MEAs) by first selecting a sub-network of the most active recording sites (determined as those with the highest Mean Firing Rate, MFR), then creating a binary matrix associated with the selected electrodes, and finally examining the corresponding simplicial complex, which represents the co-activation among the electrodes in the topological map.

We select the most active electrodes and bin the peak trains to reduce the data space without losing fundamental system characteristics. We choose the electrodes by applying principal component analysis (PCA) to their firing rate profiles and we define bin windows from the range between 100 and 1000 ms, with steps of 100 ms. To obtain the binary matrix from which the simplicial complex is constructed, we need to choose an appropriate threshold linked to MFR of each electrode. For this purpose, we select and compare three possible thresholds (i.e., 5xMFR, 6xMFR, 7xMFR) [2]. Finally, the resulting simplicial complexes are analyzed.

The main finding can be summarized as follows:

• by increasing the bin window size, simplicial complexes grow: in the spontaneous recordings their sizes increase uniformly, while in the evoked ones the increase is steeper and can appear suddenly, indicating a possible two-state condition for the stimulus-evoked recordings;

• by taking into account only the electrodes with a high Coefficient of Variation (CV) of the Interspike Interval (ISI), full simplicial complexes are already obtained with small bin window sizes, indicating a single state condition (almost complete co-activation) for such bursting electrodes.

These results show that the nature of interactions among electrodes (i.e., group of neurons) is different between spontaneous and evoked activity: not only is there a shift in the bin window needed to obtain a total synchrony, but also the way it is achieved is different. It should be noted that knowing the natural behavior of a network, or a sub-network, we might introduce means to control some of its responses.

Future work will aim to eliminate the initial selection of electrodes, by including all of them or grouping neighboring electrodes without any heuristic preconditions. We would also adapt the mathematical theory recently presented in [3] to our study of co-activation and to more general functional connectivity studies. By using the software CoCoA [4], we should be able to switch from binary matrices to monomial ideals, whose canonical basis could give local information on connectivity.
